# Exploring Social Media Use Among Medical Students Applying for Residency Training: Cross-Sectional Survey Study

**DOI:** 10.2196/59417

**Published:** 2025-02-21

**Authors:** Simi Jandu, Jennifer L Carey

**Affiliations:** 1 Department of Emergency Medicine, University of Massachusetts T.H. Chan School of Medicine, Worcester, MA, United States

**Keywords:** social media, residency recruitment, Instagram, Reddit, medical students, student, residency, residency training, social media engagement, training programs, social media usage, cross-sectional survey, survey, residency training program, thematic analysis

## Abstract

**Background:**

Since the COVID-19 pandemic, residency candidates have moved from attending traditional in-person interviews to virtual interviews with residency training programs. This transition spurred increased social media engagement by residency candidates, in an effort to learn about prospective programs, and by residency programs, to improve recruitment efforts. There is a paucity of literature on the effectiveness of social media outreach and its impact on candidates’ perceptions of residency programs.

**Objective:**

We aimed to determine patterns of social media platform usage among prospective residency candidates and social media’s influence on students’ perceptions of residency programs.

**Methods:**

A cross-sectional survey was administered anonymously to fourth-year medical students who successfully matched to a residency training program at a single institution in 2023. These data were analyzed using descriptive statistics, as well as thematic analysis for open-ended questions.

**Results:**

Of the 148 eligible participants, 69 (46.6%) responded to the survey, of whom 45 (65.2%) used social media. Widely used social media platforms were Instagram (19/40, 47.5%) and Reddit (18/40, 45%). Social media influenced 47.6% (20/42) of respondents’ opinions of programs and had a moderate or major effect on 26.2% (11/42) of respondents’ decisions on program ranking. Resident-faculty relations and social events showcasing camaraderie and wellness were the most desired content.

**Conclusions:**

Social media is used by the majority of residency candidates during the residency application process and influences residency program ranking. This highlights the importance of residency programs in leveraging social media usage to recruit applicants and provide information that allows the candidate to better understand the program.

## Introduction

Since the 2020‐2021 residency application cycle, the Association of American Medical Colleges and Liaison Committee on Medical Education have recommended that programs conduct virtual interviews exclusively for residency applicants [1,2]. This recommendation allows for a more equitable residency application process, as it offloads financial and time burdens from the applicant involved with traveling, the applicant pool, bias, and interview flexibility; however, having an exclusively virtual process also comes with a loss of applicants developing rapport with faculty and residents and appreciating resident camaraderie, program culture, and what resident daily life is like [1,3].

Students and residency programs have both turned toward social media to lessen this void. Residency applicants turn to social media to gather more information about residency programs, such as details about the work environment and facilities, and residency programs use social media to promote their programs and institutions and highlight their culture, personnel, and network [4-8]. This has been shown to increase the number of programs applicants can apply to [9]. Residency programs must embrace this digital shift to adapt to the postpandemic landscape and efforts to enhance diversity and equity in medical education. Thus, social media remains an important platform for residency applicants and programs alike.

Despite its widespread usage, there is a lack of information on the impact that applicants across different specialties derive from residency programs’ social media accounts. There have been single-specialty studies that have shown that social media is used by prospective applicants during the residency recruitment process, but limited studies across specialties have been performed [4,10-12]. In this study, we performed a survey across multiple specialties to elucidate the patterns of social media consumption and its influences on medical students’ selection of a residency program.

## Methods

### Population and Setting

The study population consisted of fourth-year medical students who graduated in 2023 from an allopathic medical school in Massachusetts and who participated in the residency match program during the 2022‐2023 cycle.

### Ethical Considerations

The survey was approved by the institutional review board and deemed not human research (STUDY00001121). The survey contained a description containing the risks of participation in the study, and completion of the survey implied voluntary, informed consent. No personally identifiable information was collected, and no incentives were offered.

### Survey Development and Distribution

We developed the survey based on guidelines by Artino et al [13]. After a literature review, a focus group was held to learn more about medical students’ use and opinions of social media. This information was synthesized, and survey items were created using a combination of a Likert scale, yes or no, and open-ended questions; the survey explored demographic data, the use of social media and types of platforms, preferred social media content, and the impact of social media on residency programs. The survey was reviewed by faculty and fellows and assessed for acceptability, feasibility, and content validity of survey questions. We performed cognitive interviews for the questions and then piloted and revised the survey for clarity based on user feedback from medical students. The survey had a total of 5 pages with 6 or less questions per page, and answers could be changed. The survey was then distributed to all students at our institution who graduated in May 2023.

All fourth-year medical students who graduated in 2023 at UMass Chan Medical School were eligible for the survey and emailed a link to the anonymous electronic survey (Multimedia Appendix 1). Study data were collected and managed using the Qualtrics XM platform. The survey was distributed in May 2023 and was open for 28 days. In total, 5 reminders were sent to nonrespondents and nonfinishers at 3, 7, 14, 18, and 23 days. To avoid duplicates, each participant was sent an individual link via Qualtrics.

### Outcomes Measured

The outcomes measured included demographic data; social media platform use (platforms that were used daily, platforms used for residency programs, and the influence of social media on stages of the residency application process); content posted on social media platforms (student content that was trusted, not trusted, desired, deterrents, and then helpful); nonsocial media resources used for learning about residency programs; and reasons why participants did not use social media.

### Data Analysis

We performed simple descriptive statistics for survey questions. Nominal variables were reported as percentages and frequencies. Ordinal variables were presented as percentages. Data analysis was conducted using Prism GraphPad (version 9.5.1).

We performed a thematic analysis using an inductive constructivist approach on deidentified responses to open-ended questions of fully completed questionnaires [14,15]. Coders (SJ and JLC) independently reviewed responses via open coding, systematically generating a preliminary list of codes for each question. Using methods outlined by Nowell et al [15], these were merged into concepts, and themes were generated via constant comparison, returning to raw data, and iterative modification to develop a consensus on themes.

## Results

There were 69 respondents out of 148 eligible students in our study. Of these, 5 were excluded from the analysis because of incomplete survey responses, with a completion rate of 92.8%. The median age of survey respondents was 27 years old (range 24‐39; IQR 27‐29 years). In this cohort, 42.9% identified as a man and 57.8% identified as a woman. Further, 100% of respondents matched in the 2022‐2023 cycle, and 73.4% matched in the Northeast Region. The most popular specialties were internal medicine (25%), pediatrics (15.6%), and emergency medicine (9.4%) (Table 1).

**Table 1.** Demographic characteristics.

Characteristics	Participants, n (%)
Gender	
Women	37 (57.8)
Men	27 (42.9)
Transgender or nonbinary	0 (0)
Race or ethnicity	
American Indian or Alaska Native	0 (0)
Asian	13 (20.3)
Black or African American	0 (0)
Native Hawaiian or other Pacific Islander	0 (0)
Hispanic White	2 (3.1)
Non-Hispanic White	44 (68.8)
Other-Hispanic	2 (3.1)
Multiracial-Asian or White	3 (4.7)
Specialty	
Anesthesiology	3 (4.7)
Emergency medicine	6 (9.4)
Family medicine	4 (6.3)
Internal medicine	15 (25.0)
Internal medicine—pediatrics	1 (1.6)
Neurological surgery	1 (1.6)
Neurology	1 (1.6)
Obstetrics-gynecology	5 (7.8)
Ophthalmology	2 (3.1)
Orthopedic surgery	2 (3.1)
Otolaryngology	1 (1.6)
Pathology	1 (1.6)
Pediatrics	10 (15.6)
Psychiatry	4 (6.3)
Radiation oncology	1 (1.6)
Radiation—diagnostic	3 (4.7)
Surgery—general or preliminary	3 (4.7)
Region matched	
Northeast	47 (73.4)
Southeast	1 (1.6)
West	3 (4.7)
Southwest	7 (10.9)
Midwest	6 (9.4)
Social media platforms daily use	
Discord	3 (4.7)
Facebook	20 (31.8)
Instagram	46 (73.0)
Reddit	12 (19.1)
Snapchat	18 (28.6)
Twitter (X)	10 (15.6)
TikTok	14 (22.2)

**Figure 1.**
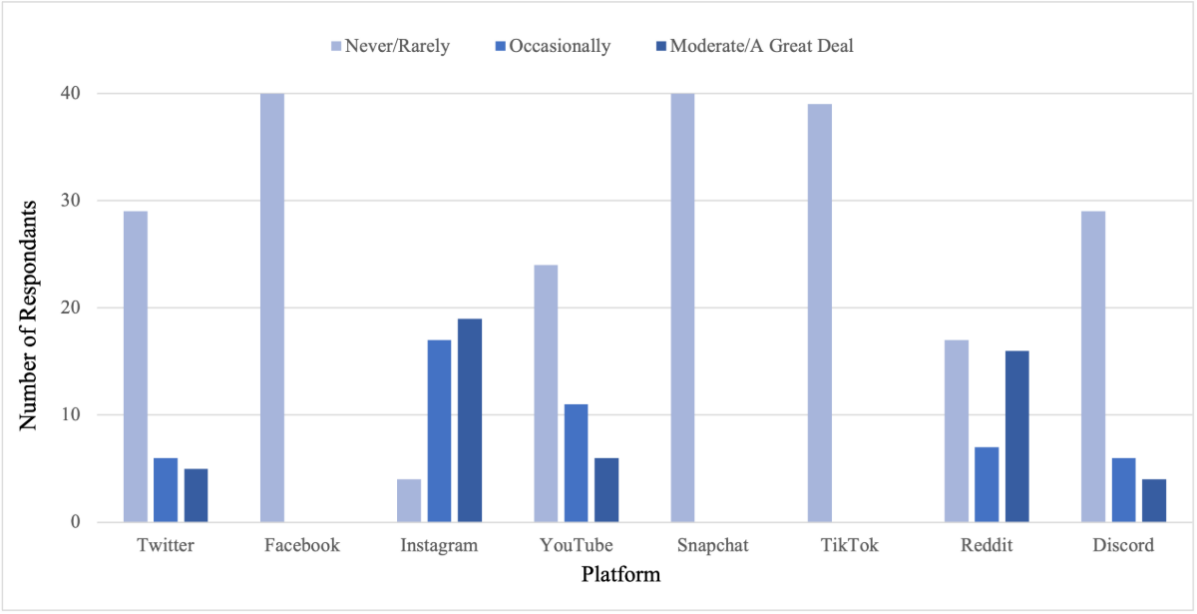
Frequency of social media platform usage when learning about residency programs.

The primary social media platform used was Instagram, with 73% (46/63) reporting daily use, followed by Facebook (20/63, 31.8%) and Snapchat (18/63, 28.6%) (Table 1). Among the respondents, 65.2% (45/69) reported using social media to learn about prospective residency programs. The most frequently used platforms for this purpose were Instagram, Reddit, and YouTube (Figure 1). Facebook, Snapchat, and TikTok were rarely or never used to learn about residency programs.

**Figure 2.**
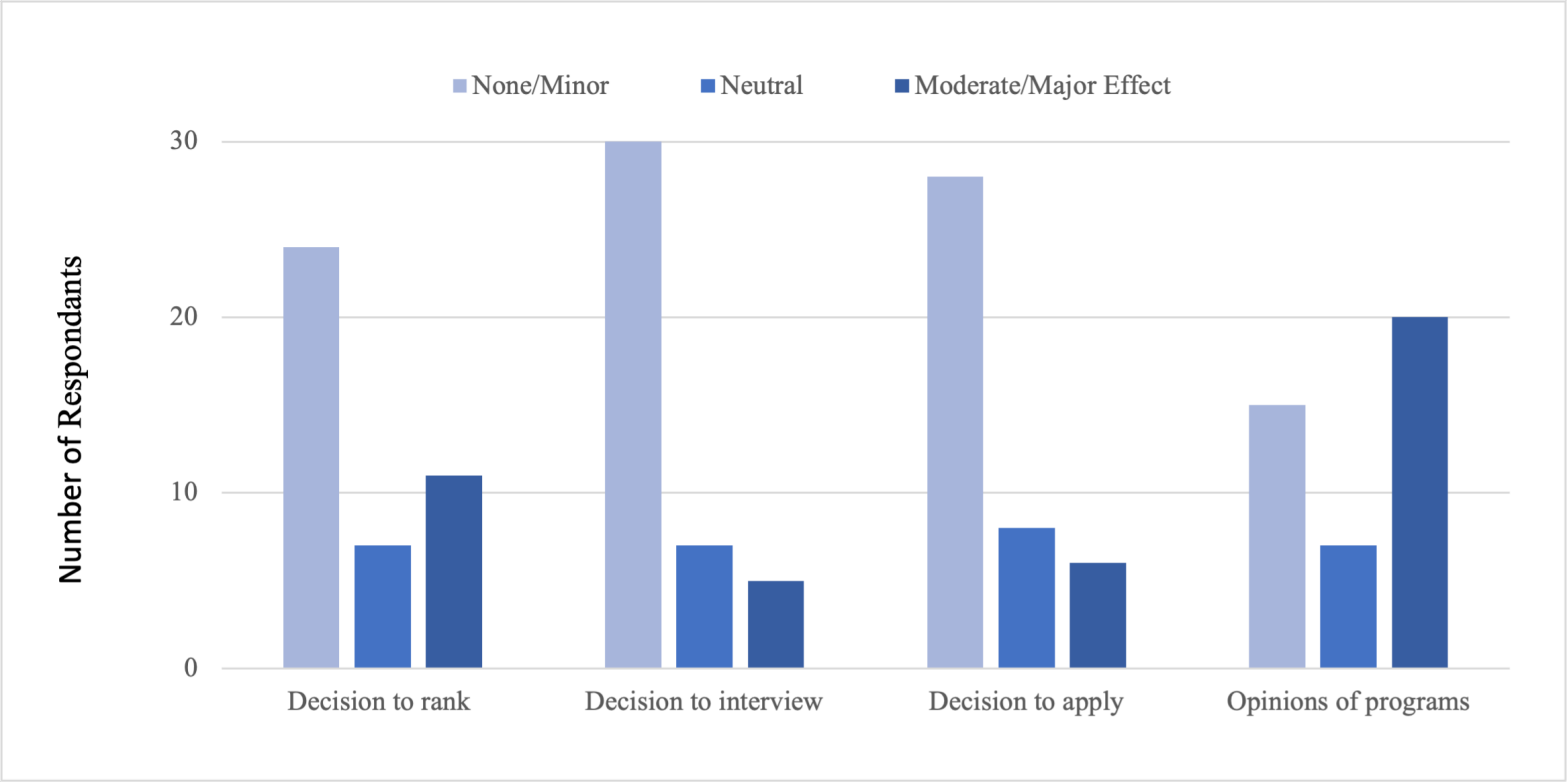
Social media influence on students’ residency application process.

Social media had a moderate or major effect on 47.6% (20/42) of respondents’ opinions about programs, while it had a lesser effect on respondents’ decision to apply (6/42, 14.3%) or interview (5/42, 11.9%) at a program. However, 26.2% (11/42) of respondents indicated that social media had a moderate or major effect on their decision to rank a program (Figure 2).

Among the 34.4% (22/64) of respondents who did not use social media, common reasons for abstaining included a perception that information could be easily distorted, social media can lead to mistrust and misrepresentation of the programs, lack of personal social media accounts, and limited usefulness or applicability of information resulting from the variable quality of social media posts. The sources trusted were the official program website, Fellowship and Residency Electronic Interactive Database Access (FREIDA), word of mouth, and personal contacts (Table 2).

**Table 2.** Resources used and reasons that students did not use social media.

	Themes
Resources used	Program website FREIDA^a^ Word of mouth Current or past residents Institutional contacts
Reasons that students did not use social media	Mistrust of social media Programs could be misrepresented Irrelevant and not applicable postings that were unhelpful Posts were not authentic Respondents did not have or use social media personally and professionally Variability in posts

a FREIDA: Fellowship and Residency Electronic Interactive Database Access.

The content deemed most valuable was resident and faculty relations (89.5%), followed by social events (86.8%) and education (60.5%) (Table 3). Thematic analysis revealed that students were attracted to posts emphasizing camaraderie, diversity, resident wellness, a genuine representation of the program’s personality, and program and curriculum information. Participants particularly valued “Day in the Life” posts that visually depicted people, facilities, and location, as well as content that focused on personal experiences or resident wellness, highlighted unique attributes of the program, and provided information on the application process (Table 4).

**Table 3.** Desired social media content when researching residency programs.

Content	Participants, n (%)
Social events	33 (86.8)
Research	13 (34.2)
Didactics	12 (31.6)
Education	23 (60.5)
Resident and faculty relations	34 (89.5)
Other	2 (5.3)

**Table 4.** Themes and quotes of content that students deemed helpful, attractive, deterrents, trusted, and not trusted.

Themes		Quotes
Content that attracted students to a program		
	Camaraderie among residents and faculty; diversity of the program	“Seeing the residents spending time together and enjoying it, even if they were posting them working on the floor together”
	Resident wellness	“Camaraderie evident on posts”
	Content invoking a genuine feeling and showcasing its personality; informational content about the program and curricula	“Multiple social events with residents of all classes, photos with attendings and residents together”
Content that students deemed helpful in learning more about programs		
	Visualizing people, facility, and location; showcasing program’s unique features; informational posts on the program; “Day in the Life”; resident wellness including what they do in their time off; personal experiences about a program; focus on advocacy	“When it was active, showed personality of programs/residents”
Content that deterred students		
	Minimal to no social media posts; lack of representation of multiple resident and faculty; negative personal anecdotes	“Less activity or presence on social media left an impression of overworked residents who didn’t have time to post, or programs with less wellness/bonding activities to show off”
	Perception of ingenuine postings	“Too much of one person”
	Lack of types of content: photos and resident highlights	“No photos of residents and attendings”
Content trusted by students		
	Personal endorsements; resident-driven content; perspectives from residents and applicants; anonymous online platforms	“Reddit, student doctor network, because people post anonymously and be honest about the negative aspects of their program”
Content not trusted by students		
	Curated social media posts	“Social media posts are curated, and I don’t trust that it’s a true reflection of the day-to-day or vibe of the program”
	Program websites; promotional videos	“The promo videos for each program on the website because you can highlight very small parts.”
	Nonanonymous content (subject to bias)	“Statements about the quality of the program, if they are happy, etc. I would not trust because the residents know the recordings will be posted. Hard to be honest when you can’t be anonymous.”
	Reddit, Discord, and chat/discussion boards (subject to bias)	“Chat/discussion boards due to potential for bias”
	Content from individuals outside the program; content from nonprogram accounts	“Message boards like Reddit I consider less reliable as anyone could share their experience with a good versus bad interview, I find these sites are very polarizing good or bad.”

In a thematic analysis among all participants, anonymous digital platforms such as Reddit and Discord were considered trustworthy by some (n=3), although others perceived them as subject to bias and could be polarizing (n=15). Respondents also reported that nonanonymous content could also be subject to bias, noting that individuals posting may not want to be honest about negative aspects of a program when posting anonymously. Content from individuals outside the program and from nonprogram accounts was generally not trusted, as content could be posted and “filled with trolls,” individuals who post intentionally provocative or inflammatory content. Finally, curated social media posts, program websites, and promotional videos were also listed among content that was not trusted (n=8), as it may only highlight certain aspects of the program (Table 4).

## Discussion

### Principal Findings

Our study revealed that Instagram was the most commonly used social media platform within our cohort. Instagram has experienced the greatest growth among new residency-specific social media accounts since March 2020, and its predominant demographic characteristics are similar to those of most prospective residents [12,16-18]. It has also been cited to be the most used platform, compared to Facebook and Twitter [19].

Interestingly, although the majority of students in this cohort reported using Facebook daily or weekly, it was almost never used to learn about residency programs by our respondents. Despite Facebook being commonly cited and compared to other platforms, it was only used more than Twitter by family medicine applicants. Otolaryngology, anesthesia, and plastic surgery applicants all used Facebook less than Twitter, with all specialties citing Instagram as the most used platform [4,7,19-21]. Facebook as a platform was shown to have the least growth, the least total number of accounts across specialties, and the least utilization among most specialties in comparison to Instagram and Twitter [17,19,20]. It is unclear why students did not use Facebook to learn about residency programs, despite their overall frequent use. One possible reason is the lack of Facebook posts by residency programs. To maximize the effectiveness of their social media presence, programs might consider focusing on Instagram rather than Facebook or linking the 2 platforms, thereby reaching 2 platforms with 1 post.

We found Reddit to be the second most popular platform. Reddit has been used by anesthesia and emergency medicine applicants as sources of information but was not this highly ranked by prior studies [20,22]. Its design facilitates ease of information exchange, and its built-in anonymity affords users the opportunity to post content without fear of repercussions. Students acknowledged that while anonymity introduces the potential for bias, anonymous online chat and discussion boards still have the potential to be trustworthy sources of information. Additionally, it is worthwhile for programs to note that negative anecdotes published on Reddit or similar platforms can deter students from programs and can be seen by those without social media accounts. The increasing popularity of Reddit suggests that it is a worthwhile avenue for social media outreach during the residency application season [22].

As social media influence the residency process, respondents are affected by their opinion and rank of a program. Social media can positively influence the opinions of programs, congruent with prior urology, otolaryngology, and plastic surgery studies, with a quarter of students’ decisions affected by social media when creating their “rank list” [7,8,23]. However, as compared to Naaseh et al [9], who found 74% of respondents increased the number of programs they applied to due to social media, we found no significant effect when applying to programs found in our study despite the positive overall impression of the program in our study along with anesthesia, general surgery, and family medicine [4,9,11,20]. Based on studies, social media can influence opinion and rank of a program, which may ultimately change where a student matches for residency and whether a residency program is able to fill all its residency positions.

Regardless of surgical or nonsurgical specialty, posts that showcase resident and faculty relations, social events, and educational material are seen as the most desired content. As seen in our study, applicants desire a sense of camaraderie and resident wellness where “the residents are spending time together and enjoying it even if they were posting them working on the floor together” [19,21]. Consistent with prior studies, applicants are interested in the resident life in and outside of the hospital [19]. “Day in the Life” posts, where residents showcase a typical working day, can help students understand what their day-to-day life will be like at a particular program. They can also help to showcase aspects of the program that are difficult to show within the virtual interview setting, such as personnel interactions, diversity, and wellness [11,21]. Finally, they can be an adjunct to highlight specific program information, including curricula, electives, rotations, research, conferences, and even interview-specific information. These are all aspects sought by students in their evaluation of a program’s social media presence and can be leveraged in the recruitment of residency candidates.

Importantly, social media can also have a negative influence on prospective applicants. Our study shows that social media accounts that do not consistently post or save content can leave the “impression of overworked residents who did not have time to post, or programs with less wellness or bonding activities to show off,” consistent with prior investigations [24]. Negative anecdotes and comments left on anonymous platforms by single individuals, although possibly isolated, nonrepresentative experiences, can have a profound negative influence on an applicant’s perception of a program, and it can be exceptionally difficult to correct these views. Programs must keep in mind that the amount and content a program posts and anonymous negative anecdotes can contribute to a negative opinion of a program, potentially affecting the application and rank process.

In our cohort, approximately one-third of the applicants did not use social media and reported using other resources. Thus, it is imperative to ensure that the official program website and Google are updated and accurate. Traditional resources include FREIDA, Doximity, word of mouth, current and past residents, and contacts within the institution [4,11,12]. These are all highly trusted content used by both social media users and nonusers.

With Instagram being the most popular social media platform, residency programs would likely benefit the most from using Instagram as their main social media platform [9,12,19,20]. Although an exact threshold is unknown, students associate less frequent Instagram posts with decreased resident wellness, and frequently posting information about residents, faculty, and program information is important for a program’s image. High-impact posts might feature a particular resident for a “Day in the Life,” social activities both inside and outside of the work environment, and highlights from resident wellness days and resident-faculty interactions. These can be categorized and saved, enabling prospective applicants to easily view them at later dates. Efforts should focus on creating authentic posts that showcase the people, diversity, and culture of the program in a fun manner while taking care to avoid professional, ethical, and legal violations [25]. Students want a glimpse of what it is like to be a resident at a particular program, and posts containing pictures and videos can enable them to see and understand the program better in the current landscape of recruitment.

### Limitations

As this is a survey-based study, the survey is subject to selection and response bias, with potential inaccuracies in the participants’ recollection of their social media usage and influence in the residency application process. However, this is the first survey across specialties to delve into the social media usage throughout their residency application process; it has to be done after Match day. We attempted to limit response bias by ensuring anonymity and distributing this survey between match day and prior to graduation to all students in this class. We had no responses that indicated they did not match and did not ask whether any applicants went through the SOAP (Supplemental Offer and Acceptance Program) process. Thus, it is possible that those likely to respond may have been those who used social media throughout the application process and those who did not have to go through the SOAP process. Further studies could look at social media use in those that matched during the NRMP (National Resident Matching Program) or the SOAP process to see if there was a difference. Questions could also directly ask about positive and negative influence to gain more information on the drawbacks of social media while being a neutral question stem.

Although the survey was developed based on the guidelines of Artino et al [13], it needs to be further validated in the future. This was a single-center study from an allopathic medical school, limiting the generalizability of the findings, as social media patterns may vary among regions and medical schools. Thus, a multi-institutional study that examines applicants’ use of social media throughout their application, interview season, and ranking process is needed to further elucidate information to be used by programs. Studies could delineate the widespread use of social media by specialties, as well as whether the applicants matched into their specialty of choice or not. Specific content that students are interested in could also be looked at for specialty (ie, procedural-based vs non–procedural-based specialties or adult vs pediatric specialties).

### Conclusions

This study offers important insights into the effects of social media on residency recruitment from the student perspective. Students use social media platforms, specifically Instagram, to make informed decisions in their residency application process; therefore, programs can use these platforms to augment their recruitment. This information can help programs develop their social media platforms to cater to their target audience and mitigate the potential negative influence of social media. With the increasing popularity of social media among this generation of applicants, its use in the residency match process is expected to increase, with the current leading social media platforms being Instagram and Reddit.

## Supplementary material

10.2196/59417 Multimedia Appendix 1 Social media survey.

## Abbreviations

FREIDA: Fellowship and Residency Electronic Interactive Database Access
NRMP: National Resident Matching Program
SOAP: Supplemental Offer and Acceptance Program
