# Supplementary material for: Exploring Social Media Use Among Medical Students Applying for Residency Training: Cross-Sectional Survey Study
Source: JMIR Med Educ. 2025 Feb 21;11:e59417. doi: 10.2196/59417 (PMC11869503; doi:10.2196/59417)
Supplement: Multimedia Appendix 1 [file mededu-v11-e59417-s001.docx]

Appendix 1: Social Media Survey - UMass Chan

Start of Block: Default Question Block

Q1 The purpose of this research is to learn more about how residency applicants use social media. To be eligible for participation, you must have applied for residency during the 2022-2023 cycle. The research will consist of a survey that takes approximately 5 minutes to complete. Participation is voluntary, and you may choose to withdraw at any time without penalty. Your responses will be kept confidential and anonymous. The results of the study may be published or presented, but individual participant data will not be shared. If you have any questions, you may contact Simi Jandu: Simi.Jandu@umassmemorial.org.

How old were you on match day in years?

▼ 20 (1) ... 70 (51)

Q3 What is your gender identity?

- Male (1)
- Female (2)
- Transgender (3)
- Non-binary (4)
- Other/Prefer not to say (5)

Q4 What is your race? Check all that apply.

- American Indian or Alaska Native (1)
- Asian (2)
- Black or African American (3)
- Native Hawaiian or other Pacific Islander (4)
- White (5)
- Other race(s) (6)

Q5 Are you Hispanic, Latino/a or of Spanish origin?

- Yes (1)
- No (2)
- Prefer not to answer (3)

Q6 What specialty did you match into?

▼ Anesthesiology (1) ... Vascular Surgery (20)

Q7 What region will you be doing residency?

- Northeast (1)
- Southwest (2)
- Southeast (3)
- West (4)
- Midwest (5)

Q8 How often do you use these social media platforms?

|  | Daily (1) | Weekly (2) | Monthly (3) | Less than Monthly (4) | Never (5) |
| --- | --- | --- | --- | --- | --- |
| Twitter (1) |  |  |  |  |  |
| Facebook (2) |  |  |  |  |  |
| Instagram (3) |  |  |  |  |  |
| Snapchat (4) |  |  |  |  |  |
| TikTok (5) |  |  |  |  |  |
| Reddit (6) |  |  |  |  |  |
| Discord (7) |  |  |  |  |  |
| Other (8) |  |  |  |  |  |

Q10 Which internet non-social media resources did you use to research prospective residency programs? (Select all that apply)

- Google (1)
- Program's website (2)
- Doximity (3)
- Reddit (4)
- Discord (5)
- Chat GPT (6)
- FREIDA (7)
- Other (8) __________________________________________________

Q11 Did you use any social media to learn more about prospective residency programs?

- Yes (1)
- No (2)

Display This Question:

If Q11 = No

Q12 If no, what did you use to learn about prospective residency programs?

________________________________________________________________

Display This Question:

If Q11 = No

Q13 Why did you not use social media?

________________________________________________________________

Display This Question:

If Q11 = Yes

Q14 If you used social media to learn about prospective residency programs, please describe how often you used each of these:

|  | Never (1) | Rarely (2) | Occasionally (3) | A Moderate Amount (4) | A Great Deal (5) |
| --- | --- | --- | --- | --- | --- |
| Twitter (1) |  |  |  |  |  |
| Facebook (2) |  |  |  |  |  |
| Instagram (3) |  |  |  |  |  |
| YouTube (4) |  |  |  |  |  |
| Snapchat (5) |  |  |  |  |  |
| TikTok (6) |  |  |  |  |  |
| Reddit (7) |  |  |  |  |  |
| Discord (8) |  |  |  |  |  |
| Other (9) |  |  |  |  |  |

Display This Question:

If Q11 = Yes

Q15 For each statement, please rank how much social media influenced:

|  | No Affect (1) | Minor Affect (2) | Neutral (3) | Moderate Affect (4) | Major Affect (5) |
| --- | --- | --- | --- | --- | --- |
| Your opinions of programs (1) |  |  |  |  |  |
| Your decision to apply to a program (2) |  |  |  |  |  |
| Your decision to interview at a program (3) |  |  |  |  |  |
| Your decision to rank a program (4) |  |  |  |  |  |

Display This Question:

If Q11 = Yes

Q9 How often did you engage with program social media posts? (ie: like, comment/reply, share/retweet, etc.)

|  | Never (1) | Rarely (2) | Occasionally (3) | A Moderate Amount (4) | A Great Deal (5) |
| --- | --- | --- | --- | --- | --- |
| Twitter (1) |  |  |  |  |  |
| Facebook (2) |  |  |  |  |  |
| Instagram (3) |  |  |  |  |  |
| Snapchat (4) |  |  |  |  |  |
| TikTok (5) |  |  |  |  |  |
| Reddit (6) |  |  |  |  |  |
| Discord (7) |  |  |  |  |  |
| Other (8) |  |  |  |  |  |

Display This Question:

If Q11 = Yes

Q16 Did social media have any negative influence on your opinions of residency programs?

- Yes (1)
- No (2)
- Not Sure (3)

Display This Question:

If Q16 = Yes

Q17 In what ways did social media have a negative influence on your opinions of residency programs?

________________________________________________________________

Display This Question:

If Q16 = Not Sure

Q18 Please describe why you are unsure if social media had a negative influence on your opinions of residency programs. 

________________________________________________________________

Display This Question:

If Q11 = Yes

Q19 What features were you looking at when viewing the residency social media account? (Select all that apply)

- Social Events (1)
- Research (2)
- Didactics (3)
- Education (4)
- Resident and Faculty Relations (5)
- Other (6) __________________________________________________

Display This Question:

If Q11 = Yes

Q20 What specific aspects of social media attracted you to programs?

________________________________________________________________

Display This Question:

If Q11 = Yes

Q21 What specific aspects of social media deterred you from programs?

________________________________________________________________

Display This Question:

If Q11 = Yes

Q22 What specific aspects of social media did you find particularly helpful?

________________________________________________________________

Display This Question:

If Q11 = Yes

Q23 How would you rate the reliability of each social media site as an information source?

|  | Very Unreliable (1) | Unreliable (2) | Neutral (3) | Reliable (4) | Very reliable (5) |
| --- | --- | --- | --- | --- | --- |
| Twitter (1) |  |  |  |  |  |
| Facebook (2) |  |  |  |  |  |
| Instagram (3) |  |  |  |  |  |
| YouTube (4) |  |  |  |  |  |
| Snapchat (5) |  |  |  |  |  |
| TikTok (6) |  |  |  |  |  |
| Reddit (7) |  |  |  |  |  |
| Discord (8) |  |  |  |  |  |
| Other (9) |  |  |  |  |  |

Display This Question:

If Q11 = Yes

Q24 Were there any aspects of social media that you were not expecting to encounter that you did?

- Yes (1)
- No (2)

Display This Question:

If Q24 = Yes

Q32 What did you encounter that you were not expecting?

________________________________________________________________

Display This Question:

If Q11 = Yes

Q25 How important did you find social media if you were unable to visit the program in person?

- Not Important (1)
- Slightly Important (2)
- Moderately Important (3)
- Important (4)
- Very Important (5)

Q26 What types of content did you trust? Why?

________________________________________________________________

Q27 What types of content did you NOT trust? Why?

________________________________________________________________

Q28 Anything else about social media that you would like to share? (Feel free to expand on anything from the above survey, comment on what aspects of social media you particularly liked or did not like, comments about any specific platforms, types of posts or anything else you think is interesting about social media use).

________________________________________________________________

End of Block: Default Question Block
